# Possible application of *Akkermansia muciniphila* in stress management

**DOI:** 10.20517/mrr.2023.81

**Published:** 2024-09-10

**Authors:** Agata Misera, Wojciech Marlicz, Albert Podkówka, Igor Łoniewski, Karolina Skonieczna-Żydecka

**Affiliations:** ^1^Department of Psychiatry, Pomeranian Medical University in Szczecin, Szczecin 71-460, Poland.; ^2^Department of Gastroenterology, Pomeranian Medical University in Szczecin, Szczecin 71-252, Poland.; ^3^Department of Biochemical Science, Pomeranian Medical University in Szczecin, Szczecin 71-460, Poland.

**Keywords:** Stress, postbiotics, gut barrier, psychiatry, microbiota

## Abstract

*Akkermansia muciniphila* (*A. muciniphila*) is a promising candidate bacterium for stress management due to its beneficial effects on the microbiota-gut-brain axis (MGBA). As a well-known mucin-degrading bacterium in the digestive tract, *A. muciniphila* has demonstrated significant benefits for host physiology. Recent research highlights its potential in treating several neuropsychiatric disorders. Proposed mechanisms of action include the bacterium’s outer membrane protein Amuc_1100 and potentially its extracellular vesicles (EVs), which interact with host immune receptors and influence serotonin pathways, which are crucial for emotional regulation. Despite its potential, the administration of probiotics containing *A. muciniphila* faces technological challenges, prompting the development of pasteurized forms recognized as safe by the European Food Safety Authority (EFSA). This review systematically examines the existing literature on the role of *A. muciniphila* in stress management, emphasizing the need for further research to validate its efficacy. The review follows a structured methodology, including comprehensive database searches and thematic data analysis, to provide a detailed understanding of the relationship between stress, microbiota, and *A. muciniphila* therapeutic potential.

## INTRODUCTION

### *Akkermansia muciniphila* mucin degradation and psychobiotic mechanisms of action

Generally, mucin degradation in the digestive tract has been considered harmful to host health. However, *Akkermansia muciniphila* (*A. muciniphila*), a mucus-degrading bacterium of human gut microbiota, benefits one’s physiology^[1]^. An increasing amount of evidence indicates that this bacterium has beneficial systemic effects on host health, primarily by enhancing immunological and metabolic functions, making it a promising potential probiotic^[2]^. Recent clinical and preclinical research has demonstrated that *A. muciniphila* also plays a crucial role in various neuropsychiatric disorders by affecting the host brain through the microbiota-gut-brain axis (MGBA)^[3]^. Recently published reviews have described that *A. muciniphila* and its metabolic products can effectively alleviate symptoms of neuropsychiatric disorders like depression, anxiety, Parkinson’s disease, Alzheimer’s disease, multiple sclerosis, strokes, and autism spectrum disorders by restoring gut microbiota, repairing the gut mucosal barrier, regulating host immunity, and modulating gut and neuroinflammation^[3,4]^. The supplementation of *A. muciniphila* boosts host metabolism, and the outer membrane protein Amuc_1100 plays a pivotal role^[5]^. Importantly, Amuc_1100 binds with Toll-like receptor 2 (TLR2) to further lower the expression of serotonin reuptake transporter (SERT) and increase 5-hydroxytryptamine (5-HT) as evidenced in Caco-2 cells and mice^[6]^. These metabolites, in turn, affect emotional states and have been linked to depression, anxiety, and potentially other psychiatric illnesses^[7,8]^. It is noteworthy that *A. muciniphila* contains not only Amuc_1100, but also so-called *A. muciniphila*-derived extracellular vesicles (EVs), which can possibly enhance its biological effects^[9]^. Additionally, EVs could play some role in mediating the psychobiotic effects of the bacterium^[10]^. EVs synthesized in psychobiotic bacteria cells were found to induce many central nervous system (CNS)-linked effects. For instance, in a study by Yaghoubfar *et al.*, a 4-week administration of EVs resulted in an increase in the serotonin pool in the colon and hippocampus in mice and in a Caco-2 cell line while decreasing it in serum^[11]^. Moreover, EVs affected the mRNA expression of genes involved in serotonin signaling and its metabolism in the colon and hippocampus and the mRNA expression of *IL-10* and *TNF-α* in the colon of the mice. The 4-week treatment is comparable to the time necessary to achieve therapeutic benefits with other antidepressants^[12]^. Notably, the metabolic benefits observed with EVs were more pronounced than those achieved with live or pasteurized *A. muciniphila* cells^[9]^. This might occur due to the increased bioavailability of active substances (and their higher concentrations), which more readily interact with host cells due to their small size and ability to cross biological barriers. This allows the bioactive components within EVs to reach target sites more efficiently than whole bacterial cells. Additionally, EVs provide a stable and protected environment for their cargo, ensuring that the bioactive molecules are delivered intact to the target cells. This stability can improve the efficacy of the therapeutic components compared to those released from whole cells, which might degrade in the gut environment^[13,14]^.

Overall, the benefits of harboring *A. muciniphila* might not only be due to mucus degradation promoting the renewal of the mucus layer and maintaining gut barrier function, but also because of the production of EVs that can interact with the host’s gut epithelium, improving gut barrier function, reducing body weight gain, and enhancing glucose tolerance. Other mechanisms that have been proposed include^[9]^:

1. Pilus-associated signaling (PAS) protein (Amuc_1100), which can interact with host immune receptors like TLR2-, enhancing gut barrier function and having immunomodulatory effects, including reducing inflammation and promoting the production of beneficial cytokines. 2. Short-chain fatty acids (SCFAs) affecting gut health by interacting with host receptors (e.g., FFAR2, FFAR3) to regulate inflammation and gut hormone release. 3. Metabolite Harmaline modulating host immune responses, enhancing bile acid signaling and reducing inflammation. 4. Glucagon-like peptide-1 (GLP-1)-inducing protein (P9) involved in regulating glucose homeostasis and energy balance. 5. Peptidoglycan muropeptides activating immune receptors such as nucleotide-binding oligomerization domain 1 (NOD1) and NOD2, contributing to gut immune responses and maintaining gut health. 6. Membrane lipids (e.g., diacyl phosphatidylethanolamine), which can activate immune receptors like toll-like receptors (TLR2-TLR1) to induce anti-inflammatory cytokine production.

These modes of action collectively contribute to the beneficial effects of *A. muciniphila* on gut health, including enhancing gut barrier function, modulating immune responses, and improving metabolic conditions.

The psychobiotic potential of *A. muciniphila* is very promising; however, due to many technological problems, the administration of probiotics containing this strain is very complicated. For this reason, technology has been developed to produce pasteurized *A. muciniphila*, which has comparable or better properties than the live bacterium and has also been recognized by the European Food Safety Authority (EFSA) as a novel food that can be safely administered to humans^[15]^. As mentioned above, there are substantial data on the potential use of *A. muciniphila* in neuropsychiatric disorders; however, little is known about the effect of this bacterium on stress specifically, which plays a crucial role in the onset of many neurobiological disorders and has recently become a critical mental health issue^[16]^. Therefore, in this narrative review, we decided to describe the relationship between stress and the microbiota. To achieve this, we aimed to analyze existing work on the effects of *A. muciniphila* on stress to organize published knowledge and guide further research on this topic.

## STRESS AND MICROBIOTA

### Stress definition and phases

Current psychological descriptions of stress are dominated by the relational view, which states that “stress is a dynamic relationship between an individual and the environment that is judged by the individual to require adaptive effort or to be beyond the ability to cope. Recognizing a relationship as stressful is determined by the subjective evaluation of the individual rather than by the objective properties of the situation”^[17]^.

Stress, in the initial phase, is a highly motivating factor leading to the activation of psychological processes, mediated by the hypothalamic-pituitary-adrenal (HPA) axis and sympathetic-adreno-medullar (SAM) axis, thus enabling effective work on a given problem. On the other hand, prolonged and more severe stress significantly decreases psychological activities. If this condition persists or if the intensity of the stress increases rapidly, a so-called destructive phase of stress occurs, in which cognitive and executive functions are impaired to a large extent^[18]^. The perception of stress is a highly subjective process, as is the individual’s stress resilience. Physiologically, however, the body’s response to stress follows specific patterns explained by biochemical pathways and processes^[19]^. With deeper analysis, it could be theorized that almost any event or stimulus can be a cause of stress subjectively. Physiological occurrences such as hunger, thirst, sleep deprivation or hormonal fluctuations can also be stressful for the body. Adding to the complexity, these stimuli are processed in different regions of the brain. As a response to a perceived stressful situation, various autonomic nervous system and emotional reactions are triggered^[20,21]^. In the scope of this review, stress is defined as a dynamic relationship requiring adaptive effort. Initial stress is motivating, but prolonged stress decreases psychological activities, leading to impaired cognitive and executive functions.

### Physiological responses to stress

A stressful stimulus triggers an immediate sympathetic nervous system response that releases adrenaline and noradrenaline from the adrenal glands. The HPA axis is activated with prolonged stress, resulting in the adrenal glands secreting cortisol. This stress hormone affects lipid, carbohydrate and protein metabolism and immune function. These biochemical and physiological changes manifest as heart palpitations, accelerated breathing, trembling hands, dry mouth, sweating, and/or intestinal reactions such as abdominal cramps, pain, and diarrhea^[22,23]^. Consequently, the body, with the help of the CNS and hormones, not only minimizes intestinal blood supply and mucus production but also purges food by inducing diarrhea and vomiting. The blood, along with the energy supply, is redirected to the muscles and brain, coordinating the cascade of the “fight or flight response”. These processes allowed humans to survive throughout evolution and continue to impact the body even though stress stimuli have changed. In stress-sensitive people, public speaking or financial troubles activate the “catastrophic program” in the brain in the same way that the threat of a wild animal attack or a flood previously did. It is well established that chronic stress affects gastrointestinal, physical, and mental health^[24]^. Examples of these manifestations are disorders of gut-brain interactions (DGBIs)^[25-27]^, including irritable bowel syndrome^[28-30]^ or functional dyspepsia^[31]^. However, the majority of chronic gastrointestinal disorders are also frequently associated with high comorbidity of psychological disorders^[32]^.

**Figure 1:**
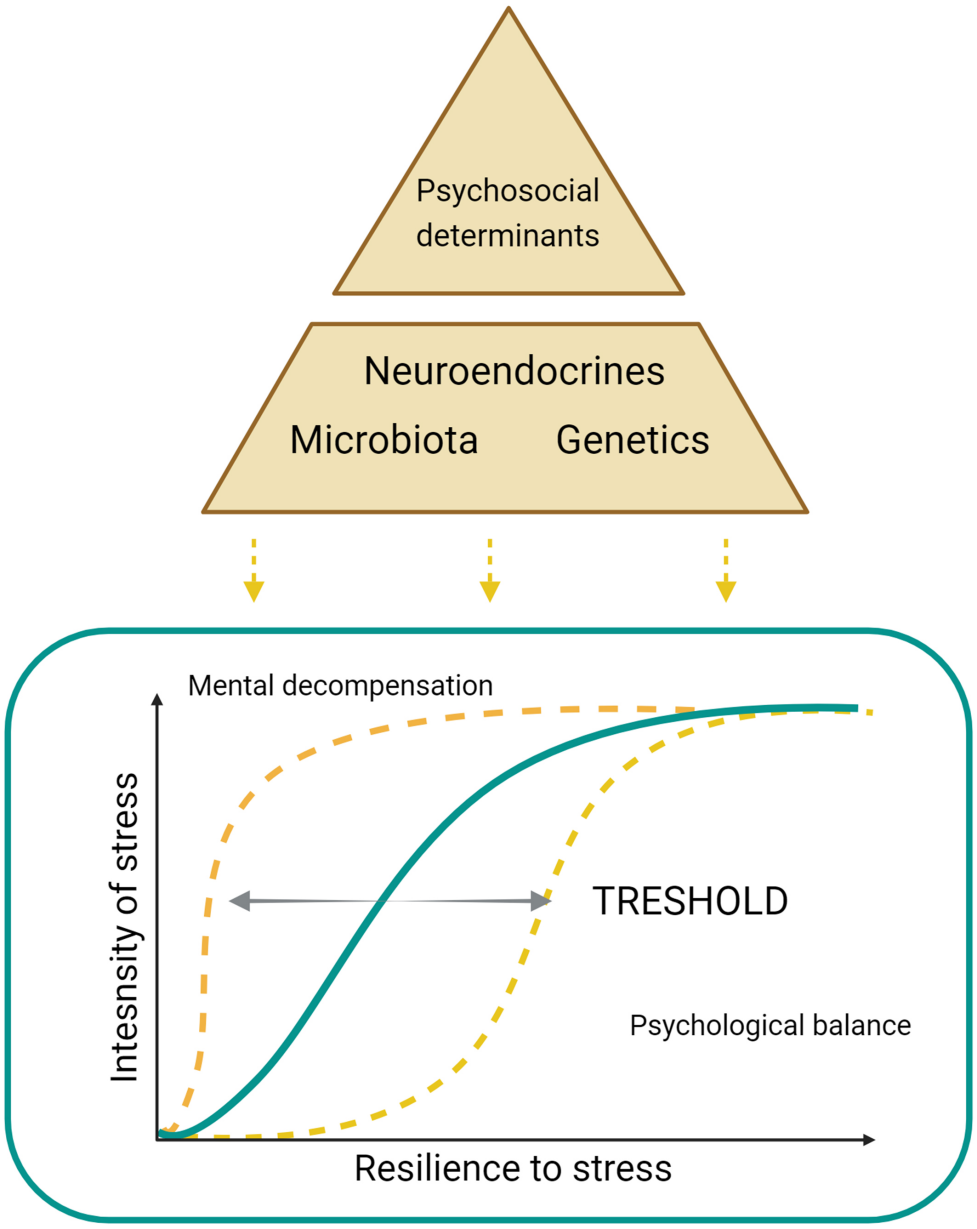
Stress vulnerability model. Created with BioRender software.

Chronic stress is one of the factors that play a role in the onset of psychiatric disorders^[33]^. This relationship is illustrated by the stress susceptibility model [Figure 1]^[34]^.

This model illustrates the psychological theory that there is a link between internal and external factors and stress susceptibility, which can impact the risk of various diseases, including mental disorders. In this theory, internal factors include genetics, biological and cellular factors, and gut microbiota composition^[35]^. External factors or buffering factors are psychosocial factors such as financial stability, a regulated lifestyle, a sense of security, or education that can impact the ability to respond to stress. These factors collectively determine how much stress an individual can endure before decompensation occurs. Decompensation manifests as atypical behavior when a person cannot regulate their emotions. It is a natural defense mechanism but can lead to mental and physical illness if persistent^[36,37]^. Standard therapies often overlook internal factors, like the gut microbiota, which are crucial for stress management. Stimuli affecting the brain are interpreted based on past experiences. If a stimulus is perceived as stressful, the brain triggers biochemical reactions, including cortisol release, impacting organs like the gut. The gut microbiota then signals the brain about the body’s state. Favorable internal factors help manage stress and prevent decompensation. Modulating these factors can break the vicious cycle and improve therapeutic outcomes^[35]^.

## MICROBIOTA AND STRESS

### Microbiota and neuromodulation

The fact that various microorganisms inhabit the gut has been known for years. However, it is recent scientific research that has begun to elucidate the functions and significance of the intestinal “microbiota”, which refers to the collection of microorganisms populating the gut. It is known that the gut microbiota is a critical element in the functioning of the brain-gut axis and that it has an impact not only on physical processes but also on mental processes. The gut microbiota plays an essential role in the human body, such that some scientists call it a separate organ^[38]^. The composition of the gut microbiota is individual to each person and is constantly changing. The number and type of microorganisms in the different levels of the gastrointestinal tract vary. Environmental conditions such as the availability of oxygen and nutrients, the pH value, the intestinal transit time, and even the structure and immunological properties of the intestinal epithelium determine the number and type of microorganisms that populate them. In addition, gender and epigenetic factors such as diet, medications, stimulants, past medical history, latitude, and age influence the formation of each individual’s gut microbiota^[39]^. Despite this, scientists are discovering more and more common traits and are gaining a better understanding of the individual microbiota and its impact on bodily function and health, both physical and mental^[40]^.

The microbiota significantly impacts the formation and modulation of the gut-brain axis^[41]^. Researchers have found that the gut microbiota is also involved in developing the hippocampus responsible for memory, the amygdala as the brain’s alarm center, and the myelination processes of prefrontal cortex neurons responsible for executive functions^[42,43]^. Furthermore, the metabolic activity of the microbiota is comparable to that of the liver. The microorganisms that make up the intestinal ecosystem break down undigested food residues by fermentation and actively and rapidly eliminate harmful antigens in the intestinal lumen. The intestinal microbiota produces vitamin K and B vitamins and, by producing hydrolase, aids in the efficient digestion of lipids. Pivotal metabolites are SCFAs, which are essential for adequately functioning of intestinal cells. Microbes stimulate the synthesis of mucins, which protect the endothelium from invasion by pathogens and toxins. In addition, they affect the CNS by producing and modulating the concentration of selected neuromediators such as serotonin, histamine, acetylcholine, norepinephrine, melatonin, and gamma-aminobutyric acid (GABA). They also affect the expression of receptors for these neurotransmitters in the brain by stimulating immune cells to release pro- and anti-inflammatory cytokines^[44]^. Furthermore, the gut microbiota impacts blood cortisol levels, affecting the functioning of various organs and exacerbating the stress spiral. Numerous scientific studies confirm the link between high levels of stress and the development of mental illnesses. Therefore, a favorable composition of the gut microbiota may be a factor that helps protect the body from mental decompensation and can contribute to one’s perception and tolerance of stress^[35]^. Overall, the gut microbiota is crucial for the brain-gut axis, affecting physical and mental processes. It influences brain development, immune responses, and the production of neuromediators and their receptors, impacting stress resilience.

### Stress-induced microbiota alterations and mental health

The significance of stress impact on the composition and function of host gut microbiota has been demonstrated in several preclinical studies. For example, a decrease in *Lactobacillus*^[45]^ and an increase in *Clostridia*^[46]^ in mice exposed to different types of stress have been documented. In contrast to studies in rodents, human studies are scarce and focus mainly on naturalistic stressors such as academic examinations or negative events, but they provide conflicting results^[47,48]^. In one of the recent studies among COVID-19 frontline healthcare workers (FHWs), experiencing stressful events has been associated with a disrupted gut microbiome for at least half a year. Microbes positively associated with good mental health were *Faecalibacterium* spp. and [*Eubacterium*] *eligens* group spp., which have anti-inflammatory properties. It was found that a low abundance of [*Eubacterium*] *hallii* group and a high abundance of *Bacteroides eggerthii* shortly after frontline work stress exposure were associated with the reappearance of posttraumatic stress symptoms^[49]^.

Numerous scientific papers also confirm the relationship between stress intensity and gut microbiota composition, a crucial part of the brain-gut axis^[50]^. It is also known that a specific composition of the gut microbiota, different from the healthy population, can be observed in mentally ill patients^[51-54]^. Thus, since stress takes part in the pathophysiology of mental illness and simultaneously has an impact on the gut microbiota, it seems essential to have an in-depth understanding of the gut microbiota as potential diagnostic and therapeutic options in case of mental disorders. Of note, psychiatric pharmacotherapy causes significant shifts in gut microbiome balance^[55]^, which further increases the risk of cardiometabolic disorders. The problem becomes further magnified as these effects are one of the main reasons for discontinuing treatment. Consequently, a high risk of recurrence of symptoms and a renewed disintegration of life might occur^[35,55]^.

Scientists have acknowledged the importance of microbiota balance in maintaining the integrity of the intestinal barrier and overall well-being. Animal experiments provided essential data on how stressful events affect the gut barrier integrity^[56-58]^. On the other hand, microbiota maintains gut barrier integrity and affects the blood-brain barrier (BBB). Microbial alterations, frequently called dysbiosis, might impair the BBB^[59]^. Stress-induced microbiota alterations lead to allostatic disturbances. The term allostasis was formed to explain the physiological adaptation mechanisms to environmental stressors, which were regulated by various mediators, including hormones, neurotransmitters, oxidative stress markers, and inflammatory processes. The short-term mobilization of allostatic mechanisms enables counteraction of negative outcomes of stress. However, chronic activation of these mediators [allostatic load (AL)] harms human health. Allostatic overload leads to negative physical and emotional outcomes and has been associated with mental and somatic disorders, including metabolic and cardiovascular diseases. It was proposed that an imbalance within gut microbiota might be a consequence of AL^[60,61]^.

In conclusion, stress alters gut microbiota composition, influencing mental health. Specific microbiota compositions are linked to mental illnesses, suggesting potential diagnostic and therapeutic options for mental disorders.

### Psychobiotics in stress management

Psychobiotics are defined as live organisms (probiotics) and prebiotics that, when ingested in adequate amounts, have a positive impact on the MGBA^[62,63]^. Animal studies have shown that gut microbiota modified by probiotics, antibiotics (ABX), and gnotobiosis (germ-free model) might significantly alter behavioral outcomes in rodents exposed to stress^[64,65]^. For instance, the intake of various probiotics was demonstrated to improve stress-induced anxiety and depressive-like behaviors in mice^[63]^. Therapeutic strategies aimed at modulation of the MGBA were proven effective as novel treatment practices in future psychiatric care. For instance, it has been well documented that administration of *Lactobacillus helveticus* Rosell-52 to animals exposed to stress is associated with diminished pathogen adherence to intestinal mucosa, decreased microbial translocation, and lowered synthesis of pro-inflammatory cytokines. These events have been associated with a protective effect on the limbic system altered by chronic exposure to stress^[66,67]^. In humans, such supplementation showed beneficial effects on mental health, including alleviating stress and anxiety. Clinical trials have documented that the administration of *Lactobacillus helveticus* Rosell-52 and *Bifidobacterium longum* Rosell-175 in healthy individuals has been associated with decreased stress-related gastrointestinal symptoms. The oral use of these probiotic strains was associated with better mood, lower perceived stress intensity, and decreased cortisol release^[68,69]^. Canadian Natural and Non-prescription Health Products Directorate issued the following license approvals related to psychobiotic use: a probiotic product composed of *Lactobacillus helveticus* Rosell-52 and *Bifidobacterium longum* Rosell-175: (i) aids in lowering overall symptoms of anxiety; (ii) supports emotional balance; (iii) aids in the management of stress-related gastrointestinal symptoms^[70]^.

## METHODS

The methodology for this study followed the scoping review framework initially developed by Arksey and O’Malley^[71]^, with subsequent updates^[72]^. The study was conducted in five main phases:

1. Formulating the research question: The question posed was, “What is known about gut microbiota alterations under the influence of *A. muciniphila* regarding its impact on health conditions in case of prolonged exposure to stress?” This broad question covered as many aspects of the topic as possible. 2. Identifying relevant studies: We searched PubMed and Google Scholar databases using key terms such as *A. muciniphila* AND (emotions OR psychiatric diseases OR psychopathology OR stress. Additionally, we manually reviewed references from relevant reviews discussing the overall impact of *A. muciniphila* on health. Only studies in English available by December 1, 2023, were included, with no restrictions on publication date. 3. Selecting studies: We included studies involving animals and humans that provided data on the intervention with *A. muciniphila* (live or pasteurized A or its components) to confer mental health benefits. The first and senior authors conducted the selection process over two weeks. 4. Charting the data: Data were extracted from study protocols, including stress model type, intervention, analytical techniques, key outcomes, conclusions, and limitations. 5. Collating, summarizing, and reporting the results: Data were thematically organized as paragraphs and a Table.

At the beginning of the article, we added a paragraph describing stress response in humans and the impact of stress on gut microbiota, the latter one being followed by a short communication on psychobiotics.

## *A. MUCINIPHILA* AND STRESS MANAGEMENT

### Psychobiotic potential and challenges

Live psychobiotics have certain limitations concerning their standardization and shelf-life stability^[73,74]^. The alternative is using postbiotics, which contain inactivated microorganisms and/or their components^[75]^. One novel postbiotic that interests scientists and clinicians is pasteurized *A. muciniphila*^[76]^. These bacteria are produced by anaerobic growth followed by pasteurization and freeze-drying. *A. muciniphila* is an anaerobe and Gram-negative bacterium, with an approximate gut relative abundance of 1% to 3%; however, the abundance of this bacterium is variable and can be much lower in many individuals and some may not harbor it. *A. muciniphila* subsists in the intestinal mucus layer and contacts epithelial cells at the top of villi. Intestinal mucins are a significant carbon and nitrogen source for producing SCFAs like acetate and propionate^[77]^.

Since its discovery, *A. muciniphila* has been used successfully in multiple clinical phenotypes, predominantly of metabolic origin^[78]^. Discovering the mechanism through which pasteurized *A. muciniphila* works has been the subject of several experimental studies. When pasteurized *A. muciniphila* was administered to mice, colitis-associated colorectal cancer progression was delayed *via* modulating CD8+ T cells^[79]^. Experiments suggest that a major mechanism is exerted through interacting with the host intestinal epithelial cell via the TLR2 directly with the protein Amuc_1100^[80-82]^. Pasteurized *A. muciniphila* attenuated inflammatory response in Caco-2 cell monolayers: it enhanced AMP-activated protein kinase (AMPK) activation and inhibited nuclear factor-kappa B (NF-κB)^[83]^. *A. muciniphila* and Amuc_1100 were reported to regulate the kynurenine pathway^[84]^ involved in several mental processes^[85]^. Ottman *et al.* found that Amuc_1100 significantly increased trans-epithelial electrical resistance (TEER) *in vitro*^[86]^. It has also been found that Amuc_1100 treatment in mice lowered the expression of *CNR*1, encoding the cannabinoid receptor 1 (CB1) in the jejunum^[80]^, thus increasing gut barrier integrity^[87]^. Through these studies, pasteurized *A. muciniphila* is emerging as a novel postbiotic with potential psychobiotic activity and particularly stress-management benefits.

### Review of experimental evidence

Pasteurized *A. muciniphila* was experimentally demonstrated to restore gut barrier integrity, a key element of the proper flow of signals within the MGBA^[88]^. Additionally, a body of evidence exists to link TLR signaling with stress response and psychiatric phenotypes^[89-93]^. Thus, a postulation on the potential use of *A. muciniphila* was made to reduce the negative effects of stress in animals and humans. However, a limited number of studies have aimed to assess such potential and exclusively in animal models.

One of the very first analyses was conducted by Chen *et al.* in male C57BL/6N mice, which were subjected to chronic restraint stress (CRS), an established protocol to achieve a mouse model of depression, and further given dextran sodium sulfate (DSS) to induce colitis^[94]^. In the second stage of the experiment, mice, after microbiota depletion with the ABX cocktail, received fecal microbiota transplantation (FMT) with *A. muciniphila* (1 × 10^9^ CFU/mL). The experiments showed that overall CRS induced behavioral deficits, as evaluated by open field test (OFT), tail suspension test (TST), and forced swim test (FST). It was found that DSS, but not CRS, diminished gut microbiota diversity. Nevertheless, at the phylum and genus levels, major alterations for the CRS group and in the DSS + CRS group compared to controls were found. FMT from CRS mice induced depression in recipient mice, but *A. muciniphila* implementation improved the scores in behavioral tests. In the DSS + CRS group, *A. muciniphila* supplementation elongated the colon and reduced the histopathological scores. *A. muciniphila* also improved mucosal barrier defects induced by FMT from CRS mice and rescued *MUC2* gene expression and elevated the number of goblet and MUC2-positive cells in each villus. A higher alpha diversity on the Shannon Index metric was demonstrated in mice receiving DSS and *A. muciniphila*. Notably, *A. muciniphila* increased the relative abundance of *Verrucomicrobia* and *Ruminiclostridium*. This study concluded that CRS-induced dysbiosis damages colonic mucus and induces the development of colitis along with a depressive phenotype and that *A. muciniphila* might inhibit the aggravation of colitis and improve the behavioral deficits associated with depression in mice.

Another study in an animal model by Ding *et al.* was conducted using C57BL/6 male mice aged 6-8 weeks^[95]^. Three groups of rodents were analyzed: mice subjected to CRS (3 weeks), mice not subjected to CRS, and mice treated with *A. mucinphila* after CRS (3 weeks, 5 × 10^8^ CFU/mL *via* oral gavage). After the treatment protocol, mice were subjected to behavioral tests and hippocampal biospecimens were collected to analyze hormones, neurotransmitters, and brain-derived neurotrophic factor (BDNF) RNA expression. Blood was taken to evaluate serum untargeted metabolome and hepatic and renal biochemical parameters. Additionally, cecal microbiota was analyzed using the next generation sequencing (NGS) amplicon-based technique. It was observed that mice treated with *A. muciniphila* exhibited a significant improvement in the OFT, TST, and FST behavioral tests compared to mice with CRS exposure only. In gene expression analyses, *A. muciniphila* intervention diminished the level of corticosterone release after CRS exposure, along with elevation in serum dopamine and hippocampal BDNF but not serum serotonin levels.

The differences were significant regarding the CRS exposure group. As far as gut microbiota composition is concerned, differences in structure were found (*A. muciniphila vs.* CRS-only groups: higher abundance of *Verrucomicrobia*, lower of *Epsilonbacteraeota*, *Patescibacteria*, *Chloroflexi*, and *Acidobacteria*; genus level: lower of *Helicobacter*, *Candidatus_Saccharimonas*, *Eubacterium_brachy_group*, and *Lachnoclostridium*)*.* However, the principal component analysis revealed no differences between the groups. In a functional prediction approach, pathways linked to neurodegenerative diseases were inhibited in the CRS + *A. muciniphila* compared to the CRS-only group. Additionally, the CRS + *A. muciniphila* group exhibited a lowered abundance of genes involved in tryptophan metabolism, geraniol degradation, caprolactam degradation, fluorobenzoate degradation, and Parkinson’s disease. Metabolites between all three groups clustered differently. CRS and CRS + *A. muciniphila* groups differed in metabolites linked to cholinergic synapse, fat digestion and absorption, degradation of aromatic compounds, fatty acid degradation, vitamin digestion and absorption, butanoate metabolism, carbon metabolism, pantothenate and CoA biosynthesis, metabolic pathways, and digestion and absorption. In correlation analyses, it was found that *A. muciniphila* abundance in the gut positively correlated with β-alanyl-3-methyl-l-histidine, edaravone, and 2”,3”,6”-Tris-*O*-(3,4,5-trihydroxybenzoyl)-3’-glucosyl-2’,4’,6’-trihydroxyacetophenone. In addition, a negative correlation with respect to 2-pyrrolidineacetic acid and amino pyrrolnitrin was observed. β-Alanyl-3-methyl-l-histidine and edaravone were upregulated in CRS + *A. muciniphila* group; thus, in the final part of this study, these agents were administered to CRS mice to observe the effects. In two out of three behavioral tests, this administration significantly improved the results compared to the CRS-only group. β-Alanyl-3-methyl-l-histidine lowered the corticosterone level while edaravone serum elevated serotonin concentration. Both metabolites improved hippocampal *BDNF* expression but not significantly compared to the CRS-only group. The study further supported that *A. muciniphila* can abolish the effect of CRS *via* the regulation of hormones, neurotransmitters, and BDNF levels, as well as modifications in gut microbiota and serum metabolism.

The effects of the Amuc_1100 protein, located on the outer membrane of the *A. muciniphila,* in mouse models of depression were investigated in another experiment^[96]^. Depression-like behavior was induced in C57BL/6 mice by exposure to six-week-long chronic unpredictable mild stress (CUMS), which is meant to mimic daily life stressors. Four groups were analyzed: control, CUMS, CUMS + antidepressant fluoxetine (FLX) (20 mg/kg), and CUMS + Amuc_1100 (80 µg/day). Treatments were administered by oral gavage during or after CUMS. Treatment with Amuc_1100 at both time points improved the CUMS-induced behavioral deficits significantly, which was not observed with FLX treatment, but the authors did highlight a high statistical tendency toward such. 5-HT levels in the serum and colon increased in FLX and Amuc_1100 interventions compared to CUMS-only group. When the dorsal raphe nucleus (DRN) involved in serotonin synthesis was immunohistochemically analyzed, it was revealed that both interventions elevated the process. The neurocompetent factors: BDNF, CREB1, and 5-HTR1A expression were restored following CUMS exposure when *A. muciniphila* and FLX were administered.

Similarly, the hippocampal expression of pro-inflammatory cytokines (IL-6, IL-1B, TNF-α), initially elevated by CUMS, lowered significantly in both treatment groups. CUMS diminished the diversity of gut microbiota to a large, but not significant, extent as compared to control mice. The abundance of *Bacteroidota* was lowered, and the Firmicutes increased in the CUMS group, and both FLX and Amuc_1100 interventions ameliorated these changes. The other CUMS-mediated alterations (higher counts of Clostridia and lower of Bacteroidia) were only partly and non-significantly recapitulated by FLX and Amuc_1100^[96]^.

Similarly, stress induction, in the form of CUMS, and chronic alcohol exposure were utilized in a study by Guo *et al.*^[97]^. C57BL/6J specific pathogen free (SPF) mice were treated with *A. muciniphila* (2.5 × 10^9^ CFU/200 μL) *via* oral gavage for five weeks. *A. muciniphila* improved the loss of weight post alcohol exposure, immobility time in TST, and sucrose preference in the sucrose preference test (SPT). In addition, such bacterial supplementation diminished the liver enzyme elevation induced by alcohol. Stress induced *via* alcohol and CUMS was alleviated with *A. muciniphila*, which was confirmed by decreasing immobility time in FST and TST and an elevated preference toward sucrose in the SPT. However, *A. muciniphila* did not alter the mice’s global locomotor abilities, anxiety, or fear memory. The tested intervention increased 5-HT in the prefrontal cortex and the gut, as the expression of the rate-limiting enzyme of 5-HT was not altered between *A. muciniphila* and vehicle-treated mice exposed to alcohol. On the other hand, it was decreased in the case of CUMS. Consequently, this proved that *A. muciniphila* enlarges the serotonin pool independently of this limiting bio-catalyzer. *A. muciniphila* gavage also inhibited the expression of serotonin transporter in the gut but not in the brain for both stress model mice, which can further be responsible for elevated serotonin pool. *A. muciniphila* also decreased the expression of cFos in enteric nerves, pointing out that such intervention impacts gut-to-brain signaling through these nerves. To add, a very recent study^[98]^ pointed out that modified Amuc_1100 (Amuc_1100Δ80 without 80 N-terminal amino acids with higher affinity for TLR2 as compared to Amuc_1100) in a mouse model of CUMS also upregulated a tryptophan hydroxylase 1 (Tph1) limiting serotonin expression in the gut. Furthermore, as evidenced by this group previously^[96]^, the intervention downregulated the 5-HTR1A-CREB-BDNF signal pathway and restored gut microbiota composition post CUMS exposure. Interestingly, Amuc_1100Δ80 diminished the inflammatory state and downregulated the HPA axis, consequently improving rodent depressive-like behavioral deficits.

In a study by Sun *et al.*, depressive and anxious behaviors were induced *via* an antibiotic cocktail (ABX; Ampicillin, Streptomycin, and Clindamycin;1 g/L) administration lasting three weeks^[8]^. One study group received *A. muciniphila* (1.5 × 10^9^ CFU/200 μL) per day, while the other was gavaged with the Amuc_1100 protein (100 μg/200 μL) per day, and these periods lasted two weeks. ABX was administered continuously during the intervention period. During a series of behavioral tests, it was shown that *A. muciniphila*/Amuc_1100 alleviated depression-like behavioral deficits induced by the ABX. Specifically, in an OFT, the number of entries into the center and time spent there were significantly elevated in Amuc_1100-treated mice as compared to the group treated with ABX only. The similar observation was recorded in the case of time spent in a lightbox; however, in the case of the entries into the lightbox, a significant improvement was also found for *the A. muciniphila*-treated group compared to solely ABX-treated mice. As for immobility in the TST, similar improvements were noted, but in the FST, only Amuc_1100 treatment significantly reduced the immobility time. In the case of total distance traveled in the OFT and time in open arms in the elevated plus maze (EPM) test, both treatments showed improvements; however, these were not statistically significant compared to either control (healthy, vehicle-treated mice) or ABX-treated counterparts. When gut microbiota was evaluated, it was demonstrated that ACE, Chao, and Simpson indices decreased due to ABX exposure, with the first ones being recovered with *A. muciniphila* gavage. The abundance of *A. muciniphila*, Firmicutes, *Clostridia*, and *Oscillospiraceae* was altered in the ABX group. In the group treated with *A. muciniphila*, a significantly higher abundance of Firmicutes and *Bacilli* was observed*.* On the other hand, Amuc_1100 significantly elevated *Oscillospiraceae.* At the prediction of a functional level, PICRUSt analyses showed that *A. muciniphila*/Amuc_1100 alleviated the functions of amino acid transport and metabolism, coenzyme transport and metabolism, and lipid transport and metabolism diminished with the ABX cocktail alone. Hippocampal and cortex mRNA expression analyses showed that BDNF and tropomyosin receptor kinase B (TrkB) increased in the case of *A. muciniphila*/Amuc_1100 compared to the ABX group. The latter was significantly higher in the case of Amuc_1100 compared to *A. muciniphila*. To add, markers of excessive activation of astrocytes, thus neurotoxicity, glial fibrillary acidic protein (GFAP), and cellular protooncogen c-Fos mRNA levels were restored in the hippocampus of *A. muciniphila* and Amuc_1100 groups.

Finally, 5-HT levels in serum and hippocampus were increased in the case of both interventions as compared to ABX alone. In contrast, corticosterone levels in serum and mRNA of the glucocorticoid receptor in the hippocampus decreased. The study confirmed that *A. muciniphila* and Amuc_1100 diminish antibiotic-induced anxiety and depression, affecting different parts of the GMBA, including the BDNF/TrkB signaling pathway and astrocyte activation.

The summary of the above-described studies with their limitations is arranged in Table 1. Based on these studies, it can be stated that animal studies demonstrate that *A. muciniphila* has the ability to alleviate stress-induced behavioral deficits and improve gut microbiota diversity. Mechanisms include interactions with host receptors and modulation of various stress-related pathways.

**Table 1:** Effects of *A. muciniphila* and its outer membrane protein Amuc_1100 on anxiety and depression-like behavior in mice

**Model of stress**	**Tested animals (age, weight, number, sex)**	**Intervention (dosage, duration)**	**Analytical techniques**	**Samples tested**	**Outcomes**	**Conclusions**	**Limitations**	**Ref.**
CRS and DSS-induced colitis	Male C57BL/6N mice, 18-20 g, *n* = 32 (experiment 1) and *n* = 72 (experiment 2)	*A. muciniphila* MucT (DSM 22959), 1 × 10^8^ CFU/100 µL, oral gavage for 14 days	Behavioral tests (OFT, TST, FST), ELISA, qRT-PCR, 16S rRNA gene sequencing, RNA sequencing, histology, immunohistochemistry, PAS/AB staining	Serum, colon tissue, cecal microbiota, fecal samples	Behavioral tests: increased distance in OFT, decreased immobility in TST and FST ELISA: decreased corticosterone, increased dopamine and serotonin in serum qRT-PCR: increased BDNF mRNA in hippocampus 16S rRNA gene sequencing: altered gut microbiota composition RNA sequencing: identified DEGs related to gut microbiota and colonic mucus Histology: improved colonic structure Immunohistochemistry: increased MUC2 expression PAS/AB staining: increased number of goblet cells	*A. muciniphila* supplementation protects against CRS-induced gut microbiota dysbiosis, colonic mucosal barrier damage, and aggravation of colitis	Limited to male mice; effects on females unknown Mechanisms of action not fully elucidated	Chen *et al.*, 2021^[94]^
CRS	Male C57BL/6 mice, 6-8 weeks, *n* = 6 per group	*A. muciniphila* (ATCC® BAA-835™) (1 × 10^8^ CFU/µL), daily for 3 weeks	Behavioral tests (OFT, FST, TST), ELISA, qRT-PCR, 16S rRNA gene sequencing, untargeted metabolomics	Serum, hippocampus tissue, cecal microbiota	Behavioral tests: increased distance in OFT, decreased immobility in FST and TST ELISA: decreased corticosterone, increased dopamine and serotonin in serum qRT-PCR: increased BDNF mRNA in hippocampus 16S rRNA gene sequencing: altered gut microbiota composition Untargeted metabolomics: identified 23 potential biomarkers in serum	AKK ameliorates depressive-like behavior by regulating gut microbiota, metabolites and increasing BDNF levels	Limited to male mice; female response unknown Short duration of intervention	Ding *et al.*, 2021^[95]^
CUMS	Male C57BL/6 mice, 5-6 weeks, *n* = 4-8 per group	Amuc_1100, 80 µg/200µL, daily for 6 weeks	Behavioral tests (OFT, TST, FST), ELISA, qRT-PCR, 16S rRNA gene sequencing, immunohistochemistry	Serum, colon tissue, brain (hippocampus) tissue, fecal samples	Behavioral tests: increased distance in OFT, decreased immobility in TST and FST ELISA: increased 5-HT in serum and colon, decreased corticosterone qRT-PCR: increased Tph1 in colon, increased BDNF, CREB1, 5-HTR1A in hippocampus 16S rRNA gene sequencing: improved gut microbiota diversity and composition Immunohistochemistry: increased 5-HT in DRN	Amuc_1100 improves depression-like behaviors, increases 5-HT levels in CNS and periphery, modulates gut microbiota, and upregulates BDNF and CREB1 in the hippocampus	The study was only conducted on male mice; the effects on female mice were unknown Mechanisms of MGBA interaction not fully elucidated Limited sample size for some tests Short duration of intervention	Cheng *et al.*, 2021^[96]^
CUMS	Male C57BL/6J mice, 6-8 weeks, *n* = 45	*A. muciniphila* (ATTC BAA-835) (2.5 × 10^9^ CFU/200 µL), daily for 5 weeks	Behavioral tests (OFT, FST, TST, SPT), ELISA, qRT-PCR, 16S rRNA gene sequencing, histology, immunohistochemistry	Serum, gut tissue, brain (PFC) tissue	Behavioral tests: increased distance in OFT, decreased immobility in FST and TST, increased sucrose preference in SPT ELISA: increased 5-HT in gut and PFC, decreased corticosterone qRT-PCR: increased Tph1 in gut, increased BDNF in brain 16S rRNA gene sequencing: altered gut microbiota composition Histology: improved colonic structure Immunohistochemistry: increased MUC2 expression	*A. muciniphila* alleviates depressive-like behaviors by increasing 5-HT levels in the gut and brain, modulating gut microbiota, and improving colonic structure	The study was only conducted on male mice; the effects on female mice were unknown Mechanisms of MGBA interaction not fully elucidated	Guo *et al.*, 2024^[97]^
CAE	Male C57BL/6J mice, 8 weeks, *n* = 48	*A. muciniphila* (2.5 × 10^9^ CFU/200 µL), daily for 5 weeks	Behavioral tests (OFT, FST, TST, SPT), ELISA, qRT-PCR, 16S rRNA gene sequencing, histology, immunohistochemistry	Serum, gut tissue, brain (PFC) tissue	Behavioral tests: increased distance in OFT, decreased immobility in FST and TST, increased sucrose preference in SPT ELISA: increased 5-HT in gut and PFC, decreased corticosterone qRT-PCR: increased Tph1 in gut, increased BDNF in brain 16S rRNA gene sequencing: altered gut microbiota composition Histology: improved colonic structure Immunohistochemistry: increased MUC2 expression	*A. muciniphila* alleviates depressive-like behaviors by increasing 5-HT levels in the gut and brain, modulating gut microbiota, and improving colonic structure	The study was only conducted on male mice; the effects on female mice are unknown Mechanisms of MGBA interaction not fully elucidated	Guo *et al.*, 2024^[97]^
CUMS	Male C57BL/6 mice, 5-6 weeks, *n* = 6 per group	Amuc_1100Δ80 (80 μg/day) for 3 weeks	Behavioral tests (LDB, OFT, EPM, FST, TST), ELISA, qRT-PCR, 16S rRNA gene sequencing, immunohistochemistry	Serum, colon tissue, hippocampus tissue, fecal samples	Behavioral tests: increased entries and time in light chamber (LDB), increased distance traveled (OFT), increased open arm entries (EPM), decreased immobility (FST, TST) ELISA: increased 5-HT in serum and hippocampus, decreased corticosterone qRT-PCR: increased Tph1 in colon, increased BDNF, CREB1, 5-HTR1A in hippocampus 16S rRNA gene sequencing: improved gut microbiota diversity and composition Immunohistochemistry: increased MUC2 expression	Amuc_1100Δ80 alleviates anxiety and depression-like behaviors, modulates gut microbiota, increases 5-HT levels, and enhances the 5-HTR1A-CREB-BDNF signaling pathway	The study was only conducted on male mice; effects on females are unknown Short duration of intervention Mechanisms of MGBA interaction not fully elucidated	Cheng *et al.*, 2022^[98]^
Antibiotic-induced anxiety and depression-like behavior	Male C57BL/6 mice, 6 weeks, 20 ± 2 g, *n* = 32 (Exp 1) and *n* = 72 (Exp 2)	*A. muciniphila* (1.5 × 10^9^ CFU/200 µL), Amuc_1100 (100 μg/200 µL), daily for 2 weeks	Behavioral tests (LDB, OFT, EPM, TST, FST), ELISA, qRT-PCR, 16S rRNA gene sequencing, immunohistochemistry	Serum, hippocampus, and fecal samples	Behavioral tests: increased entries and time in light chamber (LDB), increased distance traveled (OFT), increased open arm entries (EPM), decreased immobility (TST, FST) ELISA: increased 5-HT in serum and hippocampus, decreased corticosterone qRT-PCR: increased BDNF, TrkB, GFAP, c-Fos in hippocampus 16S rRNA gene sequencing: altered gut microbiota composition Immunohistochemistry: increased MUC2 expression	*A. muciniphila* and Amuc_1100 alleviate antibiotic-induced anxiety and depression by modulating the gut microbiota, increasing 5-HT levels, and affecting the BDNF/TrkB signaling pathway	The study was only conducted on male mice; the effects on females are unknown Short duration of intervention The potential impacts of antibiotic-induced gut microbiota changes on overall health are not fully explored	Sun *et al.*, 2023^[8]^

*A. muciniphila*: *Akkermansia muciniphila*; CRS: chronic restraint stress; DSS: dextran sodium sulfate; CFU: colony-forming unit; OFT: open field test; TST: tail suspension test; FST: forced swimming test; ELISA: enzyme-linked immunosorbent assay; qRT-PCR: quantitative real-time polymerase chain reaction; 16S rRNA: 16S ribosomal RNA; PAS/AB: periodic acid-Schiff and Alcian blue; BDNF: brain-derived neurotrophic factor; DEGs: differentially expressed genes; CUMS: chronic unpredictable mild stress; 5-HT: 5-hydroxytryptamine; Tph1: tryptophan hydroxylase 1; DRN: dorsal raphe nucleus; CNS: central nervous system; MGBA: microbiota-gut-brain axis; PFC: prefrontal cortex; SPT: sucrose preference test; CAE: chronic alcohol exposure; LDB: light/dark box test; EPM: elevated plus maze; TrkB: tropomyosin receptor kinase B; GFAP: glial fibrillary acidic protein, c-Fos: cellular proto-oncogene fos.

### Current human studies and applications

To the best of our knowledge, no human studies have evaluated the efficacy of *A. muciniphila* in alleviating stress manifestations. One study is ongoing: The Efficacy of Pasteurized *A. muciniphila* in Healthy Medical Workers (MENTAkHEALTH)^[99]^. Changes in the gut microbiota secondary to chronic exposure to stress can be a trigger for its manifestation in the form of somatic and psychiatric symptoms. The homeostatic disturbances and changes in the intestinal bacterial ecosystem initiate a series of inflammatory disorders in the intestine and the increased synthesis of neurocompetent metabolites, which can negatively affect the enteric nervous system (ENS) and the CNS as described earlier in this review. Medical professionals are susceptible to burnout, anxiety, and depression, but also to irregular lifestyles (e.g., shift work) and functional disorders of the digestive tract^[100]^. It was also established that an increasing number of students at medical universities abide by psychiatric or psychological therapies and thus often take anxiolytics and antidepressants^[101]^. Furthermore, research has shown that the use of antipsychotic drugs, especially of the second-generation type, can significantly affect the composition of the intestinal microbiota, increasing the ratio of Firmicutes to Bacteroidetes phyla and affecting the status of *A. muciniphila* abundance in the gut^[102]^. Another ongoing project, funded by the European Union, named Imminent Disease Prediction and Prevention at the Environment Host Interface (IMMEDIATE), aims to assess the functioning of the diet-microbiome-immunometabolism axis, which is the gateway for the initiation of the disease process. An intervention study using a so-called novel food, i.e., the pasteurized *A. muciniphila* with potent neuro- and immunocompetent properties, will evaluate the efficacy of a non-invasive therapy for the homeostasis of clinical biomarkers and the maintenance of well-being. The researchers hypothesize that administration of pasteurized *A. muciniphila* MucT (commercially available strain) at a dose of 10^10^ TFU/day for three months may favorably affect gut microbiota and increase intestinal barrier integrity, which will lead to reduced inflammation and negative health effects of stress in healthy participants.

Another very interesting application of pasteurized *A. muciniphila* MucT is reducing stress in overweight and obese individuals. A proof-of-concept clinical trial showed that pasteurized *A. muciniphila* MucT improved insulin sensitivity and reduced insulinemia and plasma total cholesterol levels.

In addition, reductions in body weight (without lifestyle or diet modifications permitted), fat mass, and hip circumference were observed compared to those of the placebo group. After three months of supplementation with this postbiotic, a reduction in blood markers of liver dysfunction and inflammation was also observed^[103]^. The relationship between stress, obesity, and diet is very complex. It has been historically known that stress disturbs cognitive processes, among them executive function and self-regulation. Stress can induce overeating (and elevates leptin, ghrelin, and neuropeptide Y), increase the propensity to choose unhealthy food, and reduce physical activity and sleep, which are critical life behaviors in maintaining health.

Finally, stress activates the HPA and impact-reward processing in the brain and the gut microbiome composition. Complicating matters further, obesity itself can be a stressful condition due to the high prevalence of weight stigma^[104]^. Therefore, pasteurized *A. muciniphila* MucT may be a valuable component of stress therapy in overweight and obese individuals. Confirmation of this thesis requires more robust clinical research.

## CONCLUSION

The role of the gut microbiota in the development of stress has been well documented and psychobiotics have gained a well-established place in its treatment. *A. muciniphila* is a Gram-negative mucus-degrading bacterium whose importance in the onset and symptomology of many diseases is increasingly well understood. Due to its action in supporting the function of the intestinal barrier, damage to which plays an essential role in the pathophysiology of psychiatric disorders, the psychobiotic properties of *A. muciniphila* provide an opportunity for novel applications and therapies. Particularly as the pasteurized bacterium has escaped the status of novel food and is finding increasing use in nutritional therapies. As described in the highlighted research in this review, the anti-stress properties of *A. muciniphila* have only been confirmed in experimental animal studies, and could depend on the Amuc_1100 protein, whose beneficial metabolic effects are well established. The beneficial role of *A. muciniphila* in stress management is promising, but further experimental and clinical studies are required to confirm its anti-stress effects.

## DECLARATIONS

### Copyright

© The Author(s) 2024.
